# Proud and fearful: Polish mothers’ emotions and motivation to maintain Polish as a HL in transnational exogamous and endogamous families

**DOI:** 10.3389/fpsyg.2025.1558750

**Published:** 2025-02-26

**Authors:** Joanna Rokita-Jaśkow, Katarzyna Panek

**Affiliations:** ^1^Department of English Studies, University of the National Education Commission, Krakow, Poland; ^2^Doctoral School, University of the National Education Commission, Krakow, Poland

**Keywords:** Polish, affect, emotions, motivation, heritage language, mothers, exogamous families, endogamous families

## Abstract

Polish is one of the most common heritage languages (HLs) in various migration contexts, which can be attributed to the motivation and commitment of Polish mothers. However, little is known about the maternal emotions and emotional strain underpinning the motivation to maintain the HL in their children raised abroad. This paper presents the results of a qualitative interview study conducted with 5 Polish mothers from transnational endogamous families and 5 from exogamous families. The qualitative content analysis of the data revealed that mothers in exogamous families experience less pressure to maintain Polish as a HL; nonetheless, in both family types, the mothers are primarily motivated to uphold the HL by feelings of pride in their cultural and linguistic heritage, along with a fear of not being able to transmit the language and identity to their children, thus failing to meet the expectations of extended family members. In endogamous families, mothers are additionally motivated by the prospect of returning and the associated possible future opportunities. The study’s findings indicate that maternal motivations and emotions arise from interactions with extended family members, which may adversely impact their agency in maintaining the HL. It is concluded that greater support for mothers in the bilingual upbringing of their children should be provided by institutions and extended family members.

## 1 Introduction

Historically, Poles emigrated for economic, political and social reasons since the 19th century, which resulted in vibrant diaspora communities around the world (Romanowski and Seretny, 2024). More recently, joining the EU in 2004, prompted many Poles to utilize opportunities for mobility in search of better life prospects abroad, often marrying and starting families abroad. As a result, Polish is frequently spoken as a minority heritage language (HL) in various non-native contexts (Kędra et al., 2021), even though it is not a language of high international recognition. In these contexts, we understand the heritage language to be the minority language spoken by immigrants of Polish descent, who often use it, at least partially, as a home language, although it is not the language officially used in the host society (Seretny and Lipińska, 2016). Recognizing its value for identity formation, parents aspire to endow their children and grandchildren with their mother tongue, who may speak it in varied degrees, from native-like to barely communicative (Polinsky and Kagan, 2007). Following this definition, Romanowski and Seretny (2024) estimate that approximately 15 million people speak some degree of Polish worldwide, having either Polish ancestry or a recent migrant background.

Maintaining the HL can be owed to dedicated mothers who aim to secure its intergenerational transmission to their children, thus acting as bridges between their homeland, the children’s grandparents, and their own children. A few recent studies on family language policy (FLP) have identified the maternal role and responsibility in the successful transmission of the HL (Connaughton-Crean, 2020; Hilbig et al., 2024; Kealy, 2019; Pedrak, 2024; Wąsikiewicz-Firlej et al., 2025; Woodward-Smith and Lorenzo-Modia, 2024). Yet, maternal emotions as drivers of motivation have not received sufficient explicit scrutiny about Polish as an HL. There is limited understanding of the emotions and emotional labor (Hochschild, 1979), experienced by the mothers who struggle with socializing their children in the new linguistic environment, on the one hand, while attempting to maintain the HL Polish, on the other. Furthermore, it is predicted that various emotions, arising from different power dynamics, can be felt in endogamous and exogamous families. Mothers’ emotional labor is also a result of the need to navigate the expectations and demands of extended family members as well as their children, which puts them under significant emotional strain. Therefore, this paper aims to fill this research gap by scrutinizing maternal emotions and motivations to maintain Polish as an HL in their children raised abroad.

## 2 Literature review: maternal emotions and motivation in maintaining HL

### 2.1 Maternal role in FLP

Mothers, as the primary caregivers for their young children, often bear the brunt of responsibility for their children’s linguistic and psycho-emotional development. Society, along with its individual members, tends to have high expectations regarding parenting styles and the desired outcomes for children. This can place a heavy burden on mothers, a phenomenon commonly referred to as “intensive mothering” (Ennis, 2015; Torsh, 2022). By the same token, in multilingual families, mothers are more likely to care for maintaining the HL in the migration setting than fathers as they seem to be more aware of the social and cultural consequences of the child not being able to communicate with the grandparents and/or the desire to hand down onto their children at least some of their home identity (Karpava, 2022; Pavlenko, 2004). Only one study (Romanowski, 2022) identified a similar role for fathers in an Australian context, indicating their willingness to actively pass on their HL to their children.

Transnational families usually wish to raise their children multilingually for they perceive the utility of the child’s multilingualism for their later life prospects. De Houwer (2015, 2020) argues that a child can develop harmonious bilingualism if two essential conditions are met: first, the family must hold a strong commitment to raising the child bilingually; second, there must be sufficient exposure to both languages to support language acquisition. This indicates that raising a bilingual child requires stamina and intentional effort from caregivers, especially mothers, from birth until mid-adolescence (De Houwer, 2015; King and Fogle, 2006; Sun, 2023).

Maintaining maternal HL can be viewed differently in various family structures. In endogenous transnational families, where both parents speak the same language and have not severed ties with their home country—often with plans to return—language maintenance may focus on preserving cultural connections. Conversely, in mixed couples (exogenous families), where one parent is from a different ethnic community and the family has chosen to live in the spouse’s home country, the approach to maintaining HL may differ due to varying cultural influences and integration into the local community (Karpava, 2022). In this situation, the mother is often the primary or sole source of input in the HL. This can create additional strain due to high expectations for her involvement in the child’s language education, which may also be linked to the emotional labor (Hochschild, 1979) she experiences.

Decisions made by parents regarding whether to continue or abandon their home language (HL) may be influenced by the support provided by their country of origin, as well as the language and educational policies of the host community. This includes the status of minority languages, which is reflected in official regulations and interactions with members of the host community, determining whether they feel accepted or excluded (Nandi and Zabrodskaja, 2024). Pułaczewska (2017) argues that parental motivations and attitudes toward maintaining a heritage language (HL) are influenced by their values. The values that parents hold shape their visions for their children’s future lives, thereby affecting their motivations for action. These values explain why individuals in similar situations may make different choices. Pułaczewska (2017, pp. 6–7) identifies several distinct value types: “*patriotic* - associated with national and ethnic identity; *religious-subjugated* to practicing Catholic religion; *collectivist*—aimed at maintaining ties with the extended family for which language is the key, *relational*—aimed at establishing a close emotional bond between the parents and the child, *caring*—eliminating possible difficulties, utilizing resources to the benefit of the child’s future, *autonomy-supportive*—respecting child attitudes and choices from the earliest days of life, and *economic-dictated* by the ease of communicating for parents (in their HL).”

Furthermore, as suggested by Hollebeke et al. (2023), family ideologies and their corresponding management plans and practices may depend on the family’s long-term goals. If a family places a high value on their HL and intends to return to their home country, they are more likely to preserve that language. In this context, more consistency in FLP can be anticipated among Polish couples temporarily emigrating abroad with the hope of returning (Rokita-Jaśkow, 2024). Therefore, it can be assumed that in mixed marriages, where the mother resides in her husband’s home country or the family lives in a country where a third language is spoken, maintaining the mother’s heritage language may be more challenging. This is due to reduced exposure to the language and the necessity for more negotiation within the family to align with the mother’s wishes.

Families employ various strategies to raise their children bilingually, e.g., adopting ‘one parent—one language’ or ‘one language—one environment’ approaches (Romaine, 1994), sending their children to HL schools (Nordstrom, 2019), or if they are unavailable, enrolling them in online HL schools (Zabrodskaja et al., 2024; Panek, 2022). For either option, they need to allocate a sufficient amount of time, finances and additional support.

This evidence indicates that, in order to successfully implement FLP in multilingual families (Schwartz, 2024; Schwartz and Verschik, 2013), mothers particularly face the challenge of balancing two desires: on the one hand, they want to help their child integrate into the host society, and on the other hand, they want to teach them their mother tongue. Raising a child in a multilingual environment presents significant challenges and dilemmas for mothers (Torsh, 2022), who may struggle to maintain the HL due to time constraints, few exposure opportunities or perceived benefits of the dominant societal language. This can lead to an emotional burden for the mothers. The emotions experienced by the mother feed back onto her motivation to maintain the HL (Beall and Tracy, 2017; MacIntyre and Vincze, 2017) as the task requires considerable effort and allocation of time and financial resources. Thus, the well-being of mothers seems to be a key prerequisite for the successful realization of FLP (De Houwer, 2020). Therefore, the emotions they experience seem to deserve further scrutiny.

### 2.2 Affect and emotions in FLP research

In language acquisition research, “affect” refers to the interconnected elements of attitudes, motivation, and emotions. Specifically, the emotions experienced while using a language or living in a particular ethnolinguistic culture influence one’s attitudes toward the speakers of that community. This, in turn, influences the motivation to learn their language. Affect has occupied a prominent place in SLA (e.g., Dewaele and MacIntyre, 2014; Gabryś-Barker and Bielska, 2013) and educational psychology research (e.g., MacIntyre and Gregersen, 2012), yet it has been considered as a factor in HL maintenance only recently (Driver and Prada, 2024; Sevinç and Mirvahedi, 2023; Song and Wu, 2024).

Emotions in multilingualism research have been studied from the socioconstructivist perspectives, indicating that emotions arise in interaction with other people and are often used interchangeably with the notion of emotionality. Following the statement of Wang et al. (2023) language emotionality refers “to emotional nature or quality in relation to language acquisition and practices” (p. 3), while emotions denote concrete feelings such as happiness, anxiety, fear etc.

According to Sevinç and Mirvahedi (2023), research on multilingualism can examine emotions in two main areas: the language of emotions and emotions related to language use. In the first area, emotions serve as a tool for socialization and building bonds between a mother and her child. Consequently, researchers typically analyze these emotions in conversational contexts to identify language choices and practices within families (e.g., Pavlenko, 2004). Secondly, emotions are examined for their interactions with multilingual acquisition and development. They are also viewed as determinants or markers of power relations between languages, identity, investment, and agency (Relaño-Pastor, 2024; Tannenbaum and Yitzhaki, 2016; The Douglas Fir Group, 2016; Pavlenko and Norton, 2007).

Emotions are expressed with language-specific vocabulary, leading to different understandings across cultures (Wierzbicka, 1999; Pavlenko, 2005; Dewaele, 2010). For example, the emotional perception of the phrase ‘Kocham Cię’ in L1 Polish as opposed to ‘I love you’ in L2 English has been found to be emotionally more profound in Polish-English adult bilinguals (Ożańska-Ponikwia, 2017). Similar perceptions of the feeling ‘tęsknota’ (longing for something) have been noted (Ożańska-Ponikwia, 2014), providing evidence that emotions are expressed more precisely and felt more intensely in bilinguals’ L1, thus serving a bond-building function. This observation may constitute an argument for the desire to use the HL in mother–child communication, at least in the first years of the child’s life, as posited by Pavlenko (2004).

Available studies on the role of parental emotions in HL maintenance point to their mediating role in shaping FLPs. For example, Wang et al. (2023) suggests that parental emotions significantly influence their ideologies, language management, and practices, which in turn affect children’s language behaviors and achievements. In her study of 12 Chinese families who immigrated to Australia with children aged 3–9, she found that parents often experience a range of negative emotions, such as anger and guilt, as they strive to ensure the transmission of their HL to their children, which is sometimes met with their children’s resentment. Parents’ attitudes are shaped by their expectations regarding the potential benefits or drawbacks for their children if they do not pass on a language that is gaining international prestige, such as Chinese. Parents have also been found to feel positive emotions such as satisfaction and pride when they successfully transmit their HL to their children.

Similarly, Hilbig et al. (2024) indicate the overwhelming guilt experienced by mothers in exogamous families who failed to teach Lithuanian as a HL to their children, stemming from judgments by other family members. The authors emphasize that this emotion arises due to the ingrained ethos of motherhood in the Lithuanian culture, which places on mothers the duty of passing down national identity to their children, closely linked to language. In another study on Lithuanian as HL, Ramonienė and Ramonaitė (2024) found that parental attitudes towards maintaining the HL varied. Both parents of endogenous couples who spoke the same HL language viewed Lithuanian as beautiful and precious. However, this perception was, on average, 20% lower when only one parent spoke the language.

Okita (2002) highlights the challenges faced by Japanese mothers married to British men who live in the UK in their efforts to maintain their HL. These mothers often carry a heavy burden as the primary homemakers, balancing the demands of British schools with the responsibility of ensuring their children continue to learn Japanese. Additionally, the author notes that the children feel overwhelmed by the extra responsibilities associated with learning the Japanese writing system. The fathers, due to their lack of proficiency in Japanese, tend to be excluded from activities that involve the Japanese language, which enhances the mother’s feeling of isolation and emotional burden.

Finally, the attitudes and relationships with extended family members may influence the mother’s and children’s attitudes towards the HL. Keeping in touch with extended family members in the home country enhances family cohesion and promotes child well-being, fostering a sense of belonging and stability that enables harmonious bilingualism to develop (Connaughton-Crean and Duibhir, 2024). By contrast, Sevinç (2022) observed that such relationships can also induce anxiety. In her study of second-generation migrant children of Turkish descent living in the Netherlands, she observed that their grandparents in Turkey were highly critical of the children’s knowledge of Turkish, considering it insufficient and incomplete. The grandparents’ view was identified to be rooted in their strong monolingual ideologies. In consequence, this stance created anxiety among the younger migrant generation and resistance to using the HL with their grandparents when in Turkey.

### 2.3 The Polish context

Since Poland’s accession to the European Union in 2004 and the increased mobility of Poles, the number of studies on FLP in post-EU accession transnational Polish families has been growing steadily (Connaughton-Crean, 2020; Jakubek-Głąb, 2024; Kealy, 2019; Kędra et al., 2021; Lubińska, 2020; Machowska-Kościak, 2020; Obojska, 2021; Pedrak, 2024; Pułaczewska, 2017; Wąsikiewicz-Firlej et al., 2025). However, the mother’s role and associated emotions in maintaining Polish as an HL have not been sufficiently scrutinized. The overview of the aforementioned studies implies that mothers in transnational families have to navigate between their parents’ (children’s grandparents) expectations and their children’s socialization needs, thus making the task of raising children in harmonious bilingualism (De Houwer, 2015; Sun, 2023) emotionally strenuous. This seems to be the case, particularly for Polish mothers, who were raised in the traditional collectivist society and who are required to conform to authority, as the older generation was. When they emigrate, they may face and socialize towards different sociocultural norms. For example, in Western European countries, the most frequently targeted by Polish migrant families, the cultural orientation is more liberal and individualistic. An attempt to socialize to L2 sociocultural norms while trying to put up with the expectations of their extended family in the home country can place their emotional stability at stake. Therefore, the goal of the following study is to shed light on these issues by answering the following questions:

1. What are the Polish mothers’ emotions and motives for maintaining Polish as an HL in exogamous and endogamous couples?
2. How does environmental support (of institutions, extended family, etc.) impact maternal motivation and emotions for maintaining Polish as an HL?

## 3 Method

The data for this study was solicited through semi-structured interviews held in Polish with Polish mothers and selected from a pool of data, constituting a larger research project investigating FLPs of Polish families living abroad. The respondents’ children have been attending the online school of Polish language and culture, mostly the Libratus school^1^. Thus, convenience and snowballing sampling were used, implying that only mothers dedicated to maintaining Polish as an HL were contacted and interviewed. The families lived in geographically diverse settings. For the purpose of the current study, the narratives of 10 mothers (M1–M10) were scrutinized. Five of these mothers were married to Poles, and the other five were married to foreigners. Table A1 provides precise information on the father’s language, place of living, family size, and family language. All mothers, except for M8, possessed higher level of education which implies a high level of language awareness. No other data about the respondents is available. To ensure anonymity, the respondents’ names have been coded and in the excerpts cited only initial letters have been used. The children’s proficiency in HL was not assessed, yet the mothers expressed satisfaction of their children’s current competence, partly due to attendance to the online school.

The interviews, each lasting 45–60 min, were conducted in Polish, recorded, transcribed, and analyzed. The extracts used in the paper were translated into English by the first author of the paper.

The study utilizes qualitative content analysis as its foundational research framework, following the methodology outlined by Krippendorff (2003). To systematically organize the data we collected, we categorized it into two distinct groups: families with endogamous structures, where partners are from similar cultural backgrounds, and families with exogamous structures, where partners come from different cultural backgrounds. After this categorization, we undertook a meticulous examination of the transcribed data. During this process, we focused on identifying initial codes that directly aligned with our research questions. In particular, we sought out and underlined specific expressions, words, and phrases that conveyed the respondents’ affective responses—namely, their attitudes, emotions, and motivations in relation to the heritage language (HL). Moreover, to enrich our analysis, we highlighted key terms and marked relevant excerpts that would not only illustrate the identified codes but also enable us to quantify their occurrences within the data set. This process allowed for a more comprehensive understanding of the emotional landscape regarding the heritage language. In accordance with ecological theory as posited by Schwartz (2024), we further investigated the origins of these emotions. This involved identifying the specific individuals or contextual factors that were associated with or responsible for the respondents’ feelings and attitudes towards the heritage language. By doing so, we aimed to provide a deeper insight into the influences shaping their affectivity, thus enhancing our overall understanding of the cultural dynamics at play.

Thirdly, we grouped the similarly sounding themes in order to generate key themes. In the end, we identified key emotions, motivations and the role of other agents in their arousal.

This paper takes a sociological and socio-constructivist approach to understanding emotions, as outlined by Oatley et al. (2006), which aligns with other research studies focused on the role of emotions in multilingualism, such as those conducted by Okita (2002) and Sevinç (2022). By viewing emotions as constructs that are learned and developed through cultural experiences, we can emphasize the significant impact that culture has on an individual’s emotional landscape. This framework allows us to delve deeper into the findings of this study, particularly regarding how cultural contexts shape maternal decision-making and child-rearing practices. For example, we explore how differences in cultural expectations, values, and practices can create emotional challenges for mothers who find themselves straddling two distinct cultures. These cultural dualities may lead to conflicts in parenting styles, feelings of inadequacy, or stress stemming from societal pressures. Finally, this approach helps to illuminate the potential emotional strains that mothers may experience when navigating the complexities of raising children in a multicultural environment. Understanding these dynamics is crucial for supporting mothers in managing their emotional health while performing the vital role of nurturing their children across cultural boundaries.

## 4 Findings

The findings are presented in relation to the key themes identified in two separate family types.

### 4.1 Motives and emotions in maintaining Polish as an HL in exogenous couples

Maintaining Polish identity in their children is a deeply felt motivation for all mothers in exogenous families. Firstly, they believe their mother tongue serves an emotional bonding function, making it easier for them to express their feelings. The decision to teach their first language to their children was often driven by practical considerations, such as staying at home and caring for the child during its early years. Speaking to their child in their native language feels more authentic to them. As M4 says,

*Talking about emotions, even using English, is like telling a story rather than telling what is inside me* (M4).

Another reason for maintaining the HL is the recognition that language is a crucial element of their identity. This understanding often deepens when individuals live abroad and see that their language serves as the only connection to their extended family in their home country, creating an intense longing for that connection. The overwhelming loneliness gives the mother time and space to reflect on her roots and identity. She feels sentimental about her background, which arouses a sense of pride. As a result, she strives to create a home environment similar to the one she experienced during her childhood. As two mothers (M3, M5) justify their willingness to speak Polish in the following way,

*When I left Poland, I felt very patriotic. I just felt a great longing for Poland, and I wanted to feel at home, and to feel that I had a Polish home* (M5).

*I guess you could call it national pride … simply put, it's sentimental…..such pride in roots, in Polishness, is priceless* (M3).

The emotion of pride contrasts with feelings of *sadness* and *fear* regarding the possibility of the child losing their heritage associated with the mother’s identity. In Polish culture there is an assumption that a mother’s identity should be passed down to her children, which appears to be an inseparable aspect of motherhood. For the mother (e.g., M5) the child losing her heritage identity is like losing the child as well. This indicates that mothers are emotionally linked to their children through the language they speak and the cultural artefacts they wish to convey, as they see the value, tradition, and cultural significance in them, as posited by sociocultural theory (Marginson and Dang, 2017; Sinha, 2024). In that respect, M5 says,

*There was a series of films about bilingual people and also stories of people who didn't have the opportunity to learn their mother's language or their father's language and who felt very bad about it. They felt as if cut off, as if incomplete … and I was very moved by that* (M5).

A few mothers (M2, M3, M5) express concerns about their children embracing the identity that comes with the dominant language of their current society. They fear that if the mother chooses to return to her home country, the child might face significant challenges in readjusting to life there, including navigating cultural differences and re-establishing connections with relatives (e.g., M5). This situation highlights the mothers’ deep awareness of the complexities involved in raising a child within a transnational family, which can sometimes jeopardise the family’s unity and sense of belonging. M2 says,

*If she chooses to do so, which I would very much like, she will be back in Poland. However, if she does not, my expectation of her is, unfortunately, that she will be Swedish* (M2).

For these mothers, the aspiration to maintain the Polish language within the family transcends mere communication; it becomes a vital thread that weaves together their identity and experiences, imparting a sense of belonging and continuity to their offspring. However, this commitment to instilling a Polish identity can trigger feelings of self-doubt when their children enter formal education and are exposed to the dominant societal language. As they watch their children navigate school, making new friends and adapting to a different linguistic environment, these mothers may struggle with fears of cultural dilution. They worry that their children’s fluency in Polish might wane or they may grow disconnected from the traditions that define their family’s legacy. As M1 says,

*Because I'm pretty a poor Polish mum myself, I mean … I've seen many, many mothers like me say that it's natural for them to speak Polish to their children, and in general, it's normal, and how could it be different? I don't know what it depends on. Anyway, I managed pretty well while J. was still relatively small. When she went to school, her French suddenly became very rich, and she suddenly became very mature linguistically; it was difficult for me to catch up on my own as if the subjects were already less life-like and more philosophical if you could talk about philosophy at the age of 4. The moment I had to ask the child a question and answer it myself, it didn't go at all. And once J. started to speak French beautifully, it got all messed up* (M1).

The mother cannot keep up with the child’s bilingual development due to lesser exposure to the societal language and, consequently, lower competency in that language.

This internal conflict—an interplay of pride in their cultural heritage and anxiety over its maintenance—highlights the complexities faced by mothers in mixed-cultural households. Their desire to nurture a sense of dual identity in their children is a formidable challenge. Mothers feel a strong sense of responsibility for maintaining their child’s heritage language (HL) for the sake of their extended family back in their home country. This language is crucial for the child to communicate with their grandparents. The child’s proficiency in the HL is evaluated during visits to the mother’s home country and is seen as a reflection of how effectively the mother has fulfilled her parenting role. Consequently, the visits appear very stressful, both for the child and the mother, and because of that, the visits do not allow for the enhancement of the child’s competence in the HL. The family members often do not realize that the mothers are solely responsible for the HL transmission, as they are the only source of HL input while living abroad. M1 gives a strong evidence of these fears,

*It was an effort on my part. It was very difficult and a lot of pressure from the Polish family. Every time we came to Poland, we just held our breath, whether J. was talking or not, and so on. Well, and you know, she felt stressed too. And she did not really want to talk, really. Well, they [grandparents] think that we are coming and that it's so easy, that it's so natural, that we're coming and we're just going to speak Polish all of a sudden, but for the children, it's actually a foreign country, because they spend maybe one week, two weeks, at the most a year there (…). So it's very difficult, and the family was also very hard on me, and in a way, very critical. And I actually had a terrible pressure that she should say something in Polish already, because God, because if she doesn't, then my brother will come right away, like: “You have children, like I saw in Bulgaria on the beach, who answer in German, I can't stand it”* (M1).

In an exogamous family, both sides of the family may put pressure on the parents regarding their decision to raise their children bilingually. They might express concerns that bilingual upbringing could lead to difficulties in school, which can add to the emotional burden on the mother. She has to endure critical remarks from external family members while trying to pursue the upbringing goals that she and her husband have set. The outcomes of these efforts may only become evident in the distant future. M5 says about her in-laws,

*At first, people tend to say that it's confusing for the child, that there is no point. My husband heard from his mother that our eldest son would have problems at school because he doesn't speak Spanish. Well, those are the opinions of people who don't know, have never been anywhere and have no idea. But now, after all these years, you can see how my son is …. how successful he is in school and beyond, and how it pays off. Well, in the beginning, we had to listen to some comments like that, unfortunately. However, we are not the only family like that. And I know others have heard it too* (M5).

The above extracts (M1, M5) illustrate that monolingual bias is a pervasive issue found in various cultural contexts. They particularly highlight how grandparents tend to prefer monolingualism, specifically in their native language. This preference may stem from a combination of cultural familiarity, historical experiences, and a desire to preserve their linguistic heritage. As a result, this generation often communicates solely in their first language, potentially limiting cross-generational interactions and the sharing of diverse linguistic practices within families.

Due to uncertain language and cultural outcomes, bilingual and bicultural parenting poses a significant dilemma. The situation becomes more complex when a mixed couple lives in a third culture, as illustrated by M2. The mother is acutely aware of the potential difficulties her family may encounter and anticipates feelings of instability and a diminished sense of belonging in their new environment. This awareness drives her to actively nurture her own cultural identity in her daughter, ensuring that her heritage remains a vital and guiding influence in their lives. M2 says,

*I don't know. I really want W. to identify in some way with Poland and the fact that she is half Polish, and without language, it would be very difficult. I think there is some value in that. Also, W. is already very culturally undefined to begin with, so if I can somehow implant that in her, then maybe she'll avoid some kind of dissonance later on* (M2).

However, not all mothers have such a pessimistic view of their children’s future. M4 views the family’s mobility as an opportunity to enrich the child’s multilingual and multicultural identity, leading to increased openness and appreciation for other cultures:

*Such openness. I think that's the most important thing: that they appreciate the tradition and culture of their family* (M4).

To conclude, mothers in exogamous families seem to view HL maintenance as an investment in their future, as they seek companionship to combat feelings of loneliness while living in their husband’s country (M1, M3). They wish to ensure that the child inherits their linguistic and cultural legacy and maintains contact with their grandparents and other family members, although achieving this goal can evoke diverse emotions. As a consequence of the latter, they tend to adopt more conciliatory perspectives, acknowledging that in the future, their child will have the right to choose which language (s) and identity they wish to embrace (M1, M3, M4, M5).

### 4.2 Emotions and motives for maintaining Polish as an HL in endogenous transnational families

In Polish couples living abroad, Polish is the primary language of communication between parents at home (cf. Table A1), though in two cases (M8 and M9), the host societal language is also used occasionally. Yet, the mothers in these families reported to insist on using only Polish in the family and reminded the fathers to adhere to this policy. With regard to the decisions about using the HL, the interviewed mothers used phrases that related to imagery and projections of the future, such as *“I cannot imagine not speaking it* ….,” (M8); *“it would have been a tragedy* (M9) …”; “*I would have felt sorry …”* (M10), which suggests a strong emotional attachment to the language the parents have been using since birth, and thus is a manifestation of their identity. It is likely, as Pavlenko (2004) after Lamendella (1977) posits, that since “the first language is acquired through perceptual and affective channels, it becomes integrated into the limbic system (…) and L1 words and phrases acquire affective connotations and become integrated with emotionally charged memories” (pp. 182–183). Thus, they consider the transmission of their HL as self-evident, especially since they have not decided to cut off ties with their families at home, consider migration as temporary and have hopes of returning. Additionally, meeting other families abroad, whose children do not speak Polish, acts as an imperative to counteract a similar situation:

*I feel a lot of emotion when I meet a lot of children of that age or older who don't speak Polish, it reinforces my motivation and the feeling that you have to act, because I can't imagine my daughter speaking like that, not having such close contact with the Polish community* (M6).

Two mothers (M8, M9) emphasize the emotion-bonding function of language, saying that the knowledge of Polish enables their children regular contact with the family at home and also constitutes an important component of their motherhood experience. Without the language, the mothers would not be able to share thoughts, emotions and experiences with their children. M8 says that understanding one another is only possible through the mother’s language. She also describes this emotion-building aspect of passing down the HL as a manifestation of love, while M10 talks about endowing the child with a language gift. M6 explains that although she could speak L2 English to her children, which she is proficient at, it is more natural for her to speak to her children in Polish, which resonates with findings of Wierzbicka’s (1999), Dewaele’s (2010), and Ożańska-Ponikwia’s (2014, 2017) research, pointing to a different meaning of emotion vocabulary in diverse cultures. She says,

*Maybe it is because I did not grow up here, but ‘I love you’ doesn't sound like ‘I love you’, but more like ‘I fell in love’; ‘I am infatuated by you’, whatever. So I can't imagine not speaking Polish* (M6).

These words show that Polish mothers in endogenous families attach a substantial emotional value to Polish as an HL and its intergenerational transmission. They also show a need to manifest and preserve the family’s identity. A strong attachment to one’s heritage can foster pride in being Polish, which closely relates to feelings of patriotism. This pride often becomes more pronounced when individuals are abroad, reflecting a deep longing for their home culture and language. For example, M7 says,

*My husband and I have literally been on the same front from the very beginning. We just really want the child to speak our mother tongue. She's only Irish because she was born here, but she's Polish by blood, not Irish at all, right?* (M7).

The desire to instill Polish identity in their children appears to be driven by a strong pride in being Polish, as well as by the expectations of their parents, who want to maintain a connection with their children and grandchildren. For these families, migration increases geographical distance but does not sever emotional ties. As a result, there is a clear imperative for the children of Polish parents to be raised within the same language and culture, regardless of where they live. Mothers play a crucial role in ensuring this intergenerational transmission, which suggests an invisible pressure placed upon them. Four mothers (M6, M7, M8, and M10) discuss this issue, expressing feelings of fear, stress regarding their parents, and a sense of impossibility in seeking forgiveness for not fulfilling this duty.

*I don't think I could, I don't know, look myself or my parents in the eyes and forgive myself for bringing them a child they couldn't talk to* (M8).

*Also, our parents and our family don't speak English, so we wanted to make sure that we didn't have situations like I heard about. It just scares me that my daughter doesn't know how to talk to her grandmother, to her grandfather, to her aunt, to her uncle. They don't know what she's saying, she doesn't know what they're saying slowly. Well, sometimes she knows, sometimes she doesn't. It just scares the hell out of me with stories like that, and I've heard these stories non-stop, and I've just seen them non-stop* (M7).

*My parents are very stressed because A. doesn't speak Polish. And even when she has those moments, when we come to Poland and she sometimes says something in English, I can see that they are anxious that she will not speak Polish. They've heard about experiences like that … . so many children's Polish just disappears, and I can't imagine that, because I also think that if she does not speak Polish, I'm taking away her relationship with her family, and I don't want that* (M6).

It is important to note that maternal fears pertain to potential future situations rather than the current reality, as discussed by Hilbig et al. (2024). These fears often involve projected feelings of shame and guilt, and therefore, make the mothers foresee and prevent them. The motivation to maintain contact with grandparents is a fundamental driving force behind maintaining HL in transnational endogamous families. For many families, the bonds with grandparents are vital to their cultural origins and familial history.

Families find innovative ways to stay connected, e.g., through messaging apps, conversations on Skype, described as emotional streaming (King-O'Riain, 2015), or frequent mutual visits, to make it easier for children to engage with their grandparents across borders. This commitment not only reinforces familial ties but also ensures that the heritage language continues to thrive, enriching the children’s understanding of their identity and cultural background. An excerpt below shows how the mother explains the need to learn the HL to her child,

*J…, “your grandparents are Polish and I cannot imagine them calling you, and you not being able to say a sentence in Polish. You have to be able to speak Polish to communicate with your family”, and I think that was my motto. That's why I put so much emphasis on the Polish language* (M10).

Their fears about how their language skills, or lack thereof, might be perceived by family members further motivate them to seek resources and tools that can enhance their children’s competence in the heritage language.

Mothers from endogamous families (M7, M9, M10) recognize the vital role that educational institutions and language professionals play in supporting the maintenance of their heritage language (HL). They feel that without this external support, they may be left as the sole providers of language input for their children, which can be daunting. Additionally, M10 experienced a profound sense of guilt for not identifying educational opportunities earlier, particularly the chance to enroll her child in an online Polish school. She often felt overshadowed by her husband, whose reluctance to prioritize these educational resources left her feeling uncertain about her instincts. This inner conflict highlighted how negative emotions and the fear of judgment can serve as powerful motivators, pushing individuals to take action to prevent unfavorable circumstances. Furthermore, it underscores the tendency of mothers to be particularly ambitious and proactive when making educational choices for their children, driven by a deep desire to provide the best possible opportunities for their growth and success.

Regarding other motivations, they are linked to a possible return to Poland and the pragmatic decisions being made in preparation for that return. Some mothers express hope about going back, as their departure was not solely their decision; rather, it was influenced by economic necessity or better job prospects for their husbands. All the interviewed parents reported this sentiment. As M10 states,

*This idea of going back, this idea of learning Polish strictly, so that they can read and write … that it would be nice to have some kind of document proving that they’ve completed Polish, came about when my husband started talking about going back to Poland permanently* (M10).

*I even thought that maybe in the future, if he decides...., I don’t know. He will know Polish very well. The conditions in Poland are very good; for example, when it comes studying. I said that he might want to come back to Poland. In that case, he will have a chance. He will also have some certificates, whether he knows Polish at all* (M9).

These mothers are convinced that learning Polish will offer their children various advantages that are not explicitly defined. These potential benefits may encompass academic opportunities in the home country, enhanced cultural understanding, and better career prospects in a globalized job market, ultimately enriching their children’s future experiences and interactions. While they acknowledge the benefits of bilingual growth for their children, their orientations are held primarily towards maintaining the HL. As for the societal language, they believe it will naturally evolve and take care of itself.

## 5 Discussion

The findings are discussed in relation to the research questions posed.

### 5.1 Polish mothers’ experienced emotions and motives for maintaining Polish as an HL in exogamous and endogamous couples

The role of mothers in maintaining HL in transnational endogamous families, i.e., those in which both parents speak Polish yet temporarily live abroad, may seem less strenuous than that of mothers living in exogamous families abroad. In the former case, the HL is typically the language of home communication. The language input does not only come from the mothers. There is more connectivity with the homeland, more frequent contact with extended family members by mutual visits, and a prospect of return, which may provide the mothers with greater motivation to cherish the HL (cf. Connaughton-Crean and Duibhir, 2024; Rokita-Jaśkow, 2024). Besides, the children have the possibility of communicating with two pairs of grandparents. By contrast, in exogenous families, the mother is the major source of HL input, although there are cases of husbands learning Polish, which provides space for translanguaging in the family (cf. Karpava, 2022).

Even though the mothers’ situations in the two types of families differ, the studied participants seem to have been driven by similar emotions. Primarily, the feeling of pride in one’s identity is felt more strongly when abroad, prompting the need to pass it on to their children as part of their identity and in building an emotional bond with their children. The HL is the tool towards this goal. Second, there is a feeling of fear, likely to turn into shame or guilt if the mothers fail the task. Feeling accountable to their children and extended family members causes a great emotional burden that they must tackle. They seem torn between the feeling of duty and pragmatic choices. While they seem to value their children’s bilingualism, they tend to prioritize the development of the HL and believe that the societal language will take care of itself. This is quite understandable, bearing in mind that the mothers are much more fluent in the HL and recognize its bond-building function. It seems good motherhood is more associated with passing the Polish language and identity than with striving to attain bilingualism in heritage and societal languages. This finding is similar to the one of Hilbig et al. (2024) among Lithuanian families who recognized that “what felt like natural mothering at the beginning transformed into a heavy-duty over time” (p. 6) as failure to transmit the language to the children would describe the mothers as “inadequate and deficient” (p. 8). We similarly recognized that “guilt has a mobilizing function” (p. 8), yet in contrast to Hilbig et al. (2024), we found that for the Polish mothers, such function and motivation was ascribed to the mere *vision* of guilt, i.e., fear that they will not conform to the expectations of others. Maternal fear had a preventative impact, as all the participants’ children were enrolled in an online Polish school. Maternal fear, therefore, impacted children’s proficiency, as all their children spoke and learnt Polish at an advanced level. The mothers did not show worry or guilt that their children do not speak the HL as in Hilbig et al.’s (2024) and Wang’s (2023) study, but that they *would not* speak it at a degree acceptable by the native speakers of Polish. It has to be acknowledged that the participants’ children were described as possessing satisfying competence in the HL for the age and school level. However, the mothers’ efforts were focused on helping them achieve perfection due to the fear that the children would be judged as inadequate in all aspects of language, such as pronunciation, vocabulary, literacy etc.

Maternal desire to maintain Polish in their children for the family’s cohesion, passing on values and maintaining contact with extended family resonates with findings in other studies (Connaughton-Crean and Duibhir, 2024; Obojska, 2021; Pedrak, 2024; Wąsikiewicz-Firlej et al., 2025). Knowing Polish is regarded as significant for the future lives of transnational children. The recognized motives for language maintenance coincide with those distinguished among Poles living in Germany by Pułaczewska (2017). They are mainly patriotic, collectivist, relational, caring and economic. This finding shows that parental motivations are first and foremost impacted by their upbringing and associated values, not the community support or status of language. Additionally, the mothers studied were motivated to maintain HL by observing other Polish families that failed to do so. These counterexamples represented a reality they could not imagine.

An explanation for such a firm standpoint on HL transmission may be rooted in Polish culture, which, until recently, could be described as predominantly collectivist and hierarchical (Czerniawska et al., 2021). This suggests that today’s parents, particularly mothers, were raised with a strong emphasis on collectivist values. As a result, they feel compelled to adhere to the hierarchy established by the older generation and follow established norms. Additionally, these mothers were raised in a monolingual and monocultural country, which likely shaped their identities in relation to nationalism, patriotism, and pride.

In the case of Polish mothers’ bilingual parenting, which caters for the development of the HL along with the societal language, is a hallmark of good parenting, as posited by King and Fogle (2006). Mothers are the key persons responsible for the transmission of HL and, therefore, feel an extreme emotional burden associated with that role. In our study, only one Spanish-speaking father (M5) in an exogamous family decided to learn Polish to keep it as a family language. In other families, paternal involvement was less visible. This finding contrasts those of Romanowski (2022), who studied Polish language maintenance in the Polish diaspora in Australia. He observed that fathers could be equally involved and responsible for HL dominance, which he explained as the changing perception of fatherhood: from breadwinning to emotional bond-building with the children. Since all fathers married English-speaking women, they could not count on their HL transmission. In that case, they assumed a role similar to that of mothers in other studies. This fact may mean that in the absence of maternal agency, the task of HL transmission can be equally undertaken by fathers.

### 5.2 The impact of environmental support (of institutions, extended family, etc.) on maternal motivation and emotions in maintaining Polish as an HL

It is important to note that the participants did not live in one community; on the contrary, they were dispersed in diverse communities and countries. This fact influences the available input in the HL and possible institutional support in the local community. The interviewed mothers were the primary sources of HL for their children. Thus, they felt a considerable burden from being the prominent persons responsible for the process and struggling to meet the expectations of extended family members, grandparents in particular. They seem to be motivated to maintain the HL by the sense of accountability to their extended family and the visions of the unacceptable possibility that their children may not be able to speak the HL. Thus, maternal motivation to maintain the HL was driven by emotions oscillating between pride and fear.

Grandparents play a significant role in raising children, particularly in collectivist societies, especially when they reside in the same household. They ensure the transmission of a code of cultural values, norms, and behavior, as evident in language and religion, to the next generations. Similarly, in migration settings, they influence the choice of the heritage language as the primary means of communication within the family (Braun, 2012; Zhu et al., 2020), they pass on cultural codes, such as politeness (Park, 2006), and teach literacy skills through reading to children (Braun, 2012).

Similarly, in case of transnational families, despite the geographical distance, grandparents maintain relationships with grandchildren through new communication methods, fostering family connectedness and cultural roots (Schuler et al., 2021; Sigad and Eisikovits, 2013), the phenomenon referred to as transnational grandparenthood (Kloc-Nowak and Timoszuk, 2021). However, the spatial mobility of parents with their children affects the maintenance of a good grandparental relationship. It demands more agency from grandparents and higher mobility at later ages (Baldassar and Merla, 2013). In a large-scale study of Polish grandparents, Kloc-Nowak and Timoszuk (2021) found that grandparents were interested in maintaining contact with their children, even if they lived abroad, which was visible in regular phone calls and visits abroad. However, the significant barrier identified was a lack of language knowledge, i.e., when neither the children speak the HL nor grandparents speak the foreign language. In such a case, grandparents may struggle with redefining their roles and feeling deprived of expected grandparenting experiences (Sigad and Eisikovits, 2013).

The fact that all study participants enrolled their children in an online Polish school suggests that they actively sought support. Online schools provide a new opportunity for uniting migrant families with their homeland and supporting HL maintenance (Zabrodskaja et al., 2024; Panek, 2022). Delegating the responsibility of educating their children in HL to professionals was particularly important as it guaranteed quality education, stability, and continuity in case the endogamous family returns to Poland, as well as stability and quality education in the case of transnational exogamous families. Institutional support was also seen as a counterbalance to the demands of the close family members (fathers) and extended family members (grandparents and parents-in-law), who were not observed to support the mothers sufficiently. Instead, they held unrealistically high expectations of the mothers maintaining the HL to a native-like degree. This finding resonates with those found by Sevinç (2022) in Turkey, i.e., the widely-held expectation that ‘a bilingual is two monolinguals in one’ (Grosjean, 1989), which can be ascribed to the similarly persistent monolingual stance among Polish society members. This stance stems from the fact that Polish society is still largely monolingual: 95% of the population speaks Polish on an everyday basis, as the 2021 census showed (GUS, 2023). Therefore they are unfamiliar with other multilingual individuals, and the phenomenon and the dynamics of multilingual competence. In this respect, we find a discrepancy between declared beliefs and overt maternal practices. In Poland, there has been a vibrant market for foreign language education from the earliest age (cf. Rokita-Jaśkow, 2025) and high recognition and aspiration for what could be regarded as elite bilingualism. Yet, when a child has an opportunity to become bilingual in a natural situation, their multilingual competence seems to be underappreciated.

All in all, in line with the ecological theory of language acquisition (Schwartz, 2024; van Lier, 2004), it can be said that that emotions arise in interactions with other members of the ecosystem. Likewise the motivation to maintain the HL is shown not to be the sole choice of the mothers themselves. Since maternal agency plays a pivotal role in HL transmission, it should be acknowledged that its activation is best possible if the mother receives appropriate support from other agents of the linguistic ecosystem at various levels, such as the extended family members and the spouse at the micro-level, the educational institutions at the meso-level, and both the host and the HL society at the macro-level (cf. Curdt-Christiansen and Huang, 2020) which can further endorse HL use through their educational and language policies. Without the support, too often, the mothers are driven by the negative emotion of fear of being inadequate, i.e., not succumbing to the expectations of the other actors of the ecology she lives in. Similarly, pride, though on the whole, seems to be a positive emotion, can bear a rule-abiding function and thus appear also as a constraint to the mother’s well-being, the lack of which defies her willingness and desire to maintain the HL.

## 6 Conclusion

The study is significant for other scholars working in the realm of maintaining Polish as an HL. To our knowledge, no other study has dealt with maternal affective tensions, experienced emotions, and the complexity of motivation behind Polish as an HL maintenance. Consequently, it may reverberate in the whole Polish community, both emigrants and in the home, showing that external pressures placed on mothers may be counteractive to their well-being and, consequently, their endurance in HL maintenance.

The findings also have educational goals: to inform the wider public, who is not accustomed to raising bilingual children, that bilingual competency never equals the competency of two monolingual native speakers of each language and varies across time and domains, and secondly, that in order to raise bilingual children, the mother needs the active support of both the family and educational institutions. Her positive mindset may help prevent the rise of negative emotions, such as feelings of judgment, allowing her to endure the formidable task of HL maintenance.

Finally, it has to be acknowledged that the study presented the emotions and motivations only of those mothers who volunteered for the study and whose children were already quite proficient in Polish as an HL, as they attended the online school of Polish. Thus the study’s limitation lies in the convenience sampling method. Little is known about mothers who placed less importance on transmitting Polish as an HL and were on the verge of failing the task, investigating which should be the goal of future studies.

## Data Availability

The raw data supporting the conclusions of this article will be made available by the authors, without undue reservation.

## Footnotes

^1^ https://www.libratus.edu.pl/

## Appendix

**Table A1:** Demographic data of the study participants.

Code	Mother’s origin	Father’s origin	Place of living	Children	Home languages	Institutional suport/online school	Length of staying abroad
M 1.	Polish	French	France	Daughter (6;0); Son (3;0)	French; Polish	Libratus	13 years
M 2.	Polish	Chinese (born in Sweden)	Sweden	Daughter (7;0)	Polish, hakka, English	ORPEG	10 years
M 3.	Polish	American	Great Britain	Son (6;0) Daughter (5;0)	Polish, English	Libratus	17 years
M 4.	Polish	Greek	Iraq	Son (14;0) Daughter (11;0) Son (9;0)	Polish, Greek, English	Libratus	15 years
M 5.	Polish	Spanish	Spain	Son (9;0) twins: Daughter (5;0) Son (5;0)	Polish, Spanish	Libratus	14 years
M 6.	Polish	Polish	USA	Daughter (5;0)	Polish	Homeschooling (enrollment to Montessori school in Poland) without online support	8 years
M 7.	Polish	Polish	Ireland	Daughter (11;0)	Polish	Libratus	17 years
M 8.	Polish	Polish	Great Britain	Son (8;0)	Polish, English	Libratus	5,5 years
M 9.	Polish	Polish	Italy	Son (8;0)	Polish, Italian	Libratus	17 years
M 10.	Polish	Polish	Great Britain	Son (12;0)	Polish	Libratus	15 years
